# Feasibility of Narrow-Band Imaging, Intraductal Biopsy, and Laser Ablation During Mammary Ductoscopy: Protocol for an Interventional Study

**DOI:** 10.29337/ijsp.180

**Published:** 2022-09-01

**Authors:** S. Makineli, M. D. Filipe, F. Euwe, A. Sakes, J. Dankelman, P. Breedveld, M. R. Vriens, P. J. van Diest, A. J. Witkamp

**Affiliations:** 1 Department of Surgical Oncology, University Medical Center, Utrecht, The Netherlands; 2 Department of Medical Technology and Clinical Physics, University Medical Center, Utrecht, The Netherlands; 3 Department of BioMechanical Engineering, Technical University, Delft, The Netherlands; 4 Department of Pathology, University Medical Center, Utrecht, The Netherlands

**Keywords:** Breast diseases, neoplasms, nipple discharge, ductoscopy, laser therapy, biopsy, minimally invasive surgery, early detection of cancer

## Abstract

**Introduction:**

Ductoscopy is a minimally invasive micro-endoscopic approach for direct visualization of intraductal lesions of the breast. Challenges of ductoscopy are low sensitivity for detecting malignancy, the lack of a proper intraductal biopsy device, and adequate treatment of intraductal lesions. This study will analyze three new approaches to enhance the effectiveness of interventional ductoscopy in patients with (premalignant) intraductal lesions: narrow-band imaging (NBI), new intraductal biopsy tools, and intraductal laser ablation. The main aims of the present study are to improve diagnostic accuracy and therapeutic efficacy of interventional ductoscopy in patients with pathological nipple discharge (PND) and to explore the feasibility of the new approaches in diagnosing and removing intraductal precursor lesions.

**Methods and analysis:**

This prospective, single-center, diagnostic feasibility study will include two patient groups. *Group A*: women with PND with no radiological suspicion for malignancy. *Group B*: women undergoing mastectomy (preventive or therapeutic). The primary endpoints for both groups are the technical feasibility of NBI ductoscopy, intraductal biopsy, and laser ablation, and as secondary endpoint the number of diagnosed and successfully treated intraductal lesions.

**Discussion:**

Enhanced ductoscopy with NBI, intraductal biopsy, and laser ablation could prevent unnecessary surgery in patients with PND.

**Ethics and dissemination:**

This study was approved by the Medical Research Ethics Committee UMC Utrecht in The Netherlands (METC protocol number 21-688/H-D). The results of this study will be published in peer-reviewed journals and presented at national and international conferences.

**Highlights:**

– Pathological nipple discharge (PND) is a common breast-related complaint in women.
– Ductoscopy, a minimally invasive technique, is used in the treatment of PND.
– This study will analyze three new approaches to enhance interventional ductoscopy of the breast: narrow-band imaging, new intraductal biopsy tools, and intraductal laser ablation in patients with (premalignant) intraductal lesions.

## Introduction

Pathological nipple discharge (PND) is one of the most common breast-related complaint [1]. PND is defined as unilateral, spontaneous, and bloody or serous discharge, usually arising from a single duct orifice of the nipple. It is often associated with breast cancer, while the most common causes of PND, ductal ectasia and intraductal papillomas, are benign [2,3]. Mammography and breast ultrasound are important imaging techniques for the detection of breast cancer. However, when PND is the only complaint, they both have limited sensitivity [4]. Magnetic resonance imaging (MRI) has shown to be a sensitive imaging technique for detecting malignancy, but specificity is low [5,6]. Therefore, the value of MRI is limited in patients with PND, and core needle biopsy or surgical excision is still necessary when MRI shows a suspicious lesion [7,8]. Because PND is regarded as a possible sign of breast cancer and standard radiologic imaging often fails to reveal the cause, most patients suffering from PND still undergo local surgical procedures (microdochectomy or major duct excision). This can lead to undesirable side effects such as scar tissue, perioperative complications, decreased sensitivity of the nipple, and compromised breastfeeding in the future [9,10,11,12,13]. Further, persistent or recurrent PND after local surgery is reported in 3 to 12% of patients [14,15]. Malignancy is found in only 5% to 8% of these operated patients [16,17,18]. This means that around 90% to 95% of the surgical procedures were performed for benign causes.

Ductoscopy is a minimally invasive micro-endoscopic technique, which allows direct visualization of the breast ducts and the possible intraductal lesions within. It can be performed under local anesthesia in the daily routine at the outpatient clinic and has proven to be safe with only a low risk of < 3% on (mild) and self-limiting complications [19,20]. Nowadays, ductoscopy is routinely used in the diagnostic work-up in patients suffering from PND [20,21]. In a previous study, ductoscopy avoided surgery in around 2 out of 3 patients with PND [22]. Ductoscopy is a developing diagnostic and interventional procedure. It has an pooled sensitivity of 58% and a pooled specificity of 92% for the diagnosis of malignancy in patients with PND with negative conventional imaging [23]. However, the current tissue extraction tool (in the form of an expandable basket) is not sufficiently accurate and only allows extraction of selected larger intraductal polypoid lesions [19,24]. Therefore, there is a need for new techniques to increase the sensitivity for the detection of premalignant lesions (especially the flat ones) and the interventional possibilities.

Firstly, to enhance the diagnostic accuracy of ductoscopy, narrow-band imaging (NBI) can be added to the procedure. NBI is an imaging technique for endoscopic diagnostic medical tests that uses a different light spectrum to mark suspicious lesions [25]. It can be electronically activated by a switch in the endoscope, leading to a peak light absorption of hemoglobin occurring at these wavelengths. Blood vessels will appear dark, allowing for improved visibility and identification of other surface structures. In gastrointestinal endoscopy, NBI has found use in identifying Barrett’s esophagus [26]. NBI is also used to identify pit patterns to classify colorectal polyps and tumors [27] and atypical dysplastic cells in the colon of patients with ulcerative colitis [28]. However, no studies have yet been conducted in which NBI is applied during ductoscopy. NBI may be useful since (pre)malignancy is known to show different patterns of vascularisation (including neovascularization and angiogenesis) compared to healthy breast tissue [29,30,31,32,33].

Secondly, to improve the interventional possibilities of a ductoscopy procedure, a cooperation between University Medical Center Utrecht and Biomechanical Engineering of the Delft University of Technology, The Netherlands, was started some years ago to develop new intraductal biopsy tools. These newly developed tools will be tested during ductoscopy for their suitability to take biopsies and accurately remove lesions. Also, adding intraductal laser ablation can be useful to enhance the interventional capacity of ductoscopy by vaporising smaller flat lesions or ablating lesion remnants after intraductal excision. Laser ablation techniques are widely used in medicine (neurosurgery, ophthalmology, head and neck surgery, and surgical urology) and have proven to be safe and able to evaporate (pre)malignant lesions [34,35]. One *ex vivo* feasibility study of endoscopic intraductal laser ablation of the breast concluded that laser ductoscopy is technically feasible and useful for intraductal interventions [36].

This study will analyze three new approaches to enhance interventional ductoscopy of the breast: NBI, new intraductal biopsy tools, and intraductal laser ablation in patients with (premalignant) intraductal lesions. The main aims of the present study are: 1) To improve diagnostic accuracy and therapeutic efficacy of interventional ductoscopy in patients with PND, and 2) to explore the feasibility of NBI, biopsy tools and laser ablation in diagnosing and treating intraductal breast cancer precursor lesions. We hypothesize that NBI will improve the diagnostic accuracy of ductoscopy as it is effective in multiple other endoscopic procedures. Also, we propose that the newly developed biopsy tools will enhance the biopsy technique, which can lead to more specific histological diagnosis and thus improvement of specificity of the ductoscopy procedure. Finally, laser ablation may improve the removal of (premalignant) intraductal lesions (or their remnants after biopsy) more precisely to enhance therapeutic efficacy.

## Methods and analysis

### Study design

This study is a phase II prospective, single-center, diagnostic feasibility trial performed in the University Medical Center in Utrecht in The Netherlands. The duration of the study will be 6–8 months of inclusion of patients. This trial starts in September 2022.

### Study population

Study subjects are selected from a clinical population from the University Medical Center Utrecht on a consecutive basis for both cohorts. This study will consist of two groups: Group A: patients with PND with no radiological suspicion for malignancy referred to the UMC Utrecht for ductoscopy, and Group B: patients undergoing mastectomy.

### Participants selection

#### Inclusion criteria

Group A: All adult women (≥18 years old) with unilateral PND and no radiological suspicion for malignancy referred to the UMC Utrecht for ductoscopy will be included.

Group B: All adult women (≥18 years old) undergoing mastectomy (preventive or therapeutic) will be included.

#### Exclusion criteria

A potential subject who meets any of the following criteria will be excluded from participation in this study for both groups:

- Pregnancy
- History of breast surgery at the affected breast wherefore ductoscopy is technically impossible
- History of radiotherapy of the breast or thorax
- Nipple retraction
- Not being able to provide written informed consent

### Sample size

No sample size calculation is performed, since this is a feasibility study. There is no comparison of outcomes. In group A, the new implementations (NBI + biopsy + laser ablation) will be performed in a maximum of 20 patients in which cannulation is possible. In group B, the new implementations will be performed in 5 patients in which cannulation is possible.

### Intervention

PND will be defined as spontaneous, single-duct nipple discharge during a non-lactating period, persisting for more than three months. Before ductoscopy, a standard diagnostic evaluation will be performed in all patients, including a complete history and physical examination and imaging (mammography, ultrasonography, and/or MRI and/or core needle biopsy) if indicated, to rule out malignancy.

**Figure 1:**
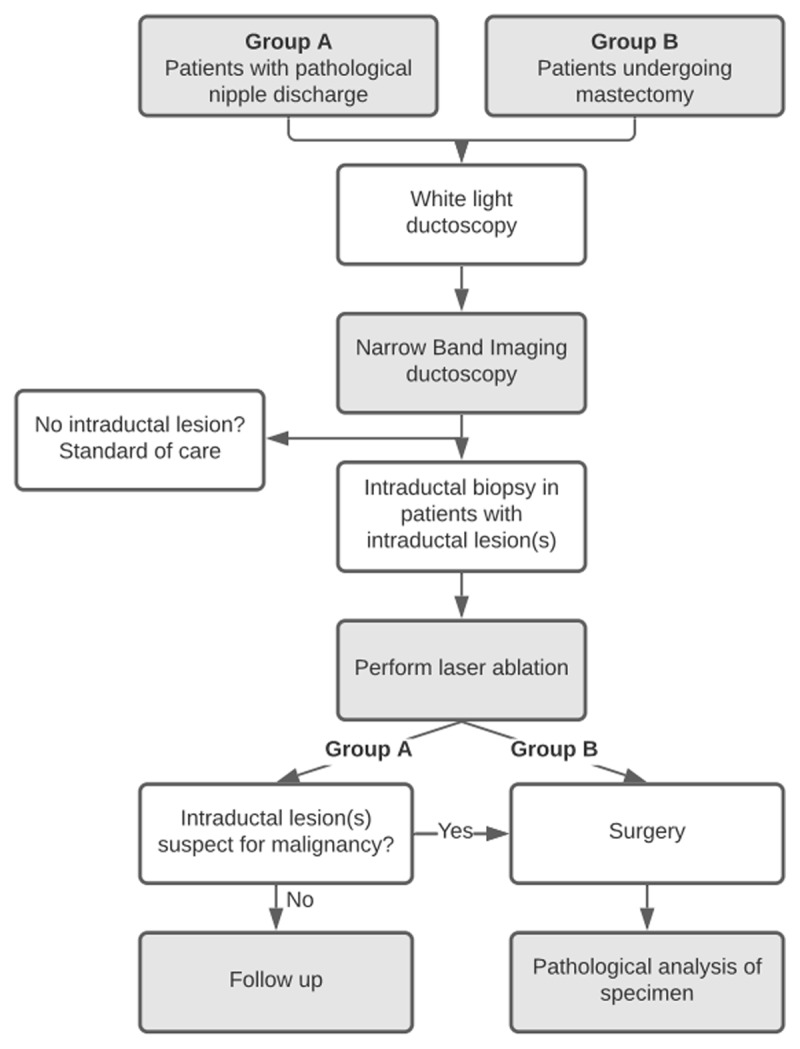
Flowchart of the study design with three new approaches added to interventional ductoscopy of the breast: narrow band imaging, tissue biopsy and laser ablation.

Patients in both groups will undergo white-light ductoscopy followed by NBI ductoscopy, as shown in the flowchart of the study design (Figure 1). Patients with intraductal lesion(s) will also undergo an intraductal biopsy with the newly designed ductoscopy tools, followed by laser ablation if indicated. Patients without intraductal lesion(s) will not undergo an intraductal biopsy or laser ablation and will be followed according to the guideline.

In group A, patients will undergo surgery depending on the outcome of ductoscopy (intraductal lesion suspicious for malignancy, persistent PND, and/or patient preference).

In group B, patients will undergo a therapeutic mastectomy (when recently diagnosed with breast cancer or DCIS) or preventive mastectomy (patients with a largely increased risk of breast cancer because of hereditary mutations in breast cancer suppression genes *BRCA1* and *BRCA2*).

Postoperatively, the surgical specimen will be histologically analyzed. The correlation between the pathological characteristics of an observed intraductal lesion in the surgical specimen and its projection in white-light/NBI/intraductal biopsy will be evaluated. Postoperative care will be according to local protocols.

### Outcome measures

#### Primary objectives

- Determine the feasibility and added value of NBI ductoscopy in diagnosing (premalignant) intraductal lesions.
- Determine the feasibility of intraductal biopsy tools for harvesting tissue of intraductal lesions.
- Determine the *in vivo* feasibility of intraductal laser ablation in patients with intraductal lesions.

#### Secondary objectives

- To compare the number of intraductal lesions found by NBI and the number found by white-light ductoscopy and intraductal biopsy (group A and B).
- Treatment success of interventional ductoscopy (biopsy and laser ablation) in treating PND (i.e., reducing the number of surgical procedures needed) (group A).
- To analyze the efficacy of intraductal biopsy and laser ablation in completely removing (premalignant) intraductal lesion(s) (group B).
- To determine the effect of interventional ductoscopy on quality of life in patients with PND (group A).

### Patient enrolment and follow-up

Study subjects will be selected from a clinical population from the University Medical Center Utrecht on a consecutive basis for both cohorts. No additional methods of recruitment of patients for inclusion will be needed.

In both groups, all study subjects eligible to be included will be asked by the treating physician if they are interested in being approached by the study coordinator for this study. The aims of the study will be explained to the patient. If subjects confirm to enter the study, informed consent will be obtained according to Good Clinical Practice guidelines.

In **group A:** The procedure will be performed in a daily routine at the outpatient clinic. The ductoscopy procedure will take 10 minutes more than usual. Lidocaine 1% will be used for local anaesthesia of the nipple. We will also ask patients to fill out questionnaires (Breast-Q, EQ-5D-5L, Ductoscopy) to analyze the effect of treatment on quality of life.

In **group B:** The ductoscopy procedure will take an additional 20 minutes and will be performed under general anesthesia directly before surgery. Postoperative care will be according to local protocols.

**Figure 2:**
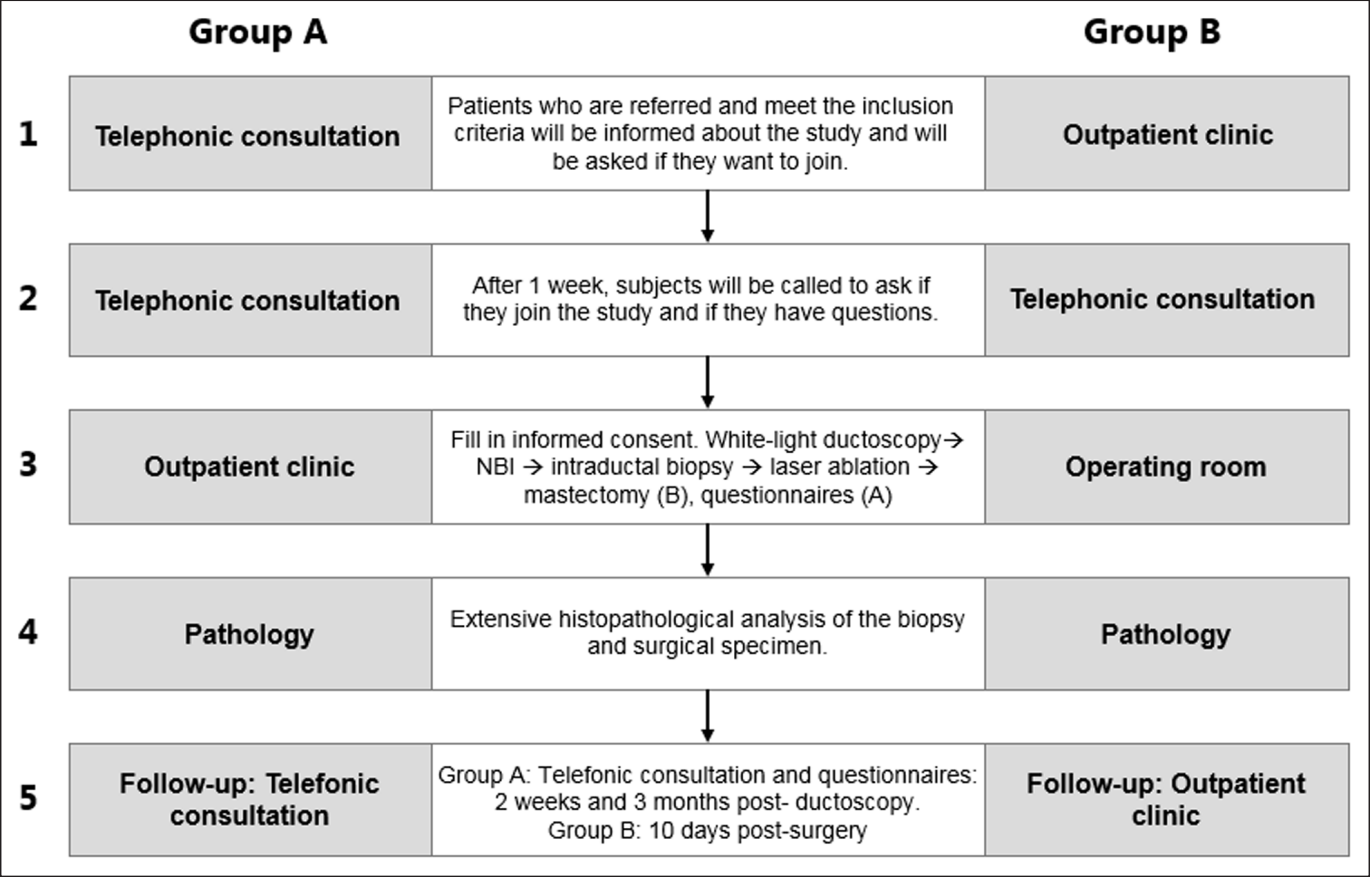
An overview of the study procedures of interventional ductoscopy enhanced by narrow band imaging (NBI), intraductal biopsy and laser ablation in patients with pathological nipple discharge (Group A) or therapeutic/preventive mastectomy (Group B).

There will be a follow-up after the procedures, as shown in Figure 2.

### Endpoints

The primary endpoints were to determine the number of intraductal lesions diagnosed by using NBI ductoscopy and treated successfully by using laser ablation. Also, to determine the ability of the new intraductal biopsy tools by performing a successful intraductal biopsy.

Secondary endpoints include the treatment success of interventional ductoscopy (biopsy and laser ablation) in treating PND. Treatment success will be achieved when symptoms disappear and nipple discharge does not return at follow-up. Furthermore, in patients who undergo surgery, to determine the accuracy of findings of NBI ductoscopy, biopsy and laser ablation during ductoscopy. Additionally, the quality of life (QOL) will be examined in patients with PND after ductoscopy.

#### Definition of a successful procedure

Successful NBI is when the ductal tree is visible during NBI ductoscopy. Successful biopsy is when it is possible to perform a biopsy with the new tools and when this tissue is sufficient to establish a correct diagnosis. Successful laser ablation is when the intraductal abnormality is no longer visible after the laser treatment. In patients who undergo surgery, the accuracy of biopsy will be determined when the biopted tissue is sufficient to establish a correct diagnosis after the final histology finding of the surgical specimen. The accuracy of laser ablation will be determined by analyzing the surgical specimen for remaining intraductal lesion.

### Statistical analysis

Descriptive statistics will be used to describe the patient and treatment characteristics of the study population. Depending on the distribution, continuous data will be described with mean and standard deviation (SD) or median and interquartile range (IQR). Differences between populations will be tested by appropriate parametric or non-parametric tests.

## Discussion

In this study, patients in both groups will undergo white-light ductoscopy, NBI ductoscopy, intraductal biopsy, and intraductal laser ablation. We defined enhanced ductoscopy as regular ductoscopy combined with NBI, improved biopsy tool, and intraductal laser ablation.

In patients with PND without radiological signs of malignancy, ductoscopy shows a sensitivity of 58% and specificity of 92% for the detection of breast cancer [23]. Additionally, ductoscopy detects (pre)cancerous lesions that were missed during regular imaging [14,37]. At the same time, MRI has a sensitivity ranging from 46 to 86% and specificity from 76 to 98% in the same patient population [38,39,40]. Nevertheless, enhanced ductoscopy might increase the diagnostic performance (with NBI and/or intraductal biopsy). The first step is to analyze the feasibility of these new approaches within this study.

Ductoscopy has already been shown to prevent unnecessary surgery in patients with PND without radiological suspicion for malignancy [22,37,41]. Papillomas are the most common cause of PND and are difficult to remove completely with current extraction tools. Therefore, this study expects that enhanced ductoscopy might improve the extraction of intraductal lesions, thereby alleviating symptoms of PND and preventing even more unnecessary surgery.
